# Adjuvant treatment in lung cancer

**Published:** 2021-04-16

**Authors:** Begoña Taboada Valladares, Patricia Calvo Crespo, Urbano Anido Herranz, Antonio Gómez Caamaño

**Affiliations:** ^1^Department of Radiation Oncology, Hospital Universitario Santiago de Compostela. Tr Choupana S/N, 15706 Santiago de Compostela, Spain; ^2^Department of Medical Oncology, Hospital Universitario Santiago de Compostela. Tr Choupana S/N, 15706 Santiago de Compostela, Spain

**Keywords:** post-operative radiotherapy, adjuvant chemotherapy, non-small-cell lung cancer, small-cell lung cancer, pathologic N2

## Abstract

**Background:**

Adjuvant treatment for both small-cell and non-small-cell lung cancer is a controversial topic. There are no published results from prospective studies that either confirm or reject the benefit of adjuvant radiotherapy, although the presentation of recent studies at a number of conferences questions whether there should be a change in the paradigm of adjuvant RT for lung cancer.

**Aim:**

The main goal of this study is to review the most relevant publications on the topic, updating the state of the matter regarding adjuvant radiotherapy following lung surgery, and analyzing the role of chemotherapy in the process.

**Relevance for patients:**

This review aims to assess the potential benefit of PORT in NSCLC and SCLC patients by looking at recent research. In doing so, it will be possible to determine which patients might benefit from it as adjuvant treatment after pulmonary resection.

## 1. Adjutancy in NSCLC

### 1.1. Introduction

Even if surgery is a standard treatment when dealing with localized non-small-cell lung cancer (NSCLC), complete resection is only achieved in 30% of the cases [1].

In 1995, a meta-analysis [2] of adjuvant chemotherapy (CT) in NSCLC comparing surgery plus CT versus surgery alone was published, effectively showing an increase in the overall survival (OS) of 5% in the CT arm, and that is why it is considered as the standard of care.

The benefits of post-operative radiotherapy (PORT) prove to be controversial as well. A 1998 meta-analysis demonstrated that the use of PORT might have a deleterious effect in Stage I-II patients, with a 2-year OS decrease of 7%. In the N2 subgroup such adverse effect was not clearly evidenced [2,3].

A correct mediastinal staging including a standardized definition of complete resection and lymphadenectomy is essential. The International Association for the Study of Lung Cancer (IASLC) [4] defines as R0 all the tumor-free margins, and as complete lymphadenectomy that which contains three intrapulmonary lymph nodes, three hilar ones, and three nodes from the N2 mediastinal region, depending on the location of the primary tumor. IASLC divides patients into three groups according to mediastinal involvement, each with different survival rates: Group 1: A single hilar lymph node station affected (N1, with a 5-year OS of 48%), Group 2: Multi-station N1 or single-station N2 (5-year OS of 35%), and Group 3: Multi-station N2 (5-year OS of 20%).

### 1.2. Adjuvant CT

The International Adjuvant Lung Cancer Trial Collaborative Group [5] designed a study to evaluate the effect of adjuvant cisplatin-based CT on complete resections of NSCLC in terms of survival rate. 1867 patients were screened between 1999 and 2000, from 148 centers in 33 countries. They were stratified into those receiving CT after surgery (932 patients) versus surgery alone (935 patients). The types of CT used were cisplatin doublets (different doses of cisplatin together with vindesine, vinblastine, vinorelbine, or etoposide). About 30.6% of the patients received PORT (1.9% pN0, 33.7% pN1, and 64.3% pN2). Both OS and disease-free survival (DFS) were higher in the CT arm. The absolute benefit in OS at 5 years was a 4.1% and in DFS, 5.1%.

In 2005 a study [6] comparing cisplatin-vinorelbine (CDDP-VNR) adjuvant CT versus observation in NSCLC patients was carried out. 482 Stage IB-II patients between 1994 and 2001 were included in the study. OS was better in the CT arm (94 m vs. 73 m, HR: 0.69, *P* = 0.04), as well as DFS (not reached vs. 46.7 m, HR: 0.60, *P* = 0.001), with acceptable toxicity.

The ANITA study [7] also compared the effects of adjuvant CDDP-VNR versus observation in 840 NSCLC S IB-IIIA patients from 101 centers. The median survival was 65.7 m in the CT arm versus 43.7 m in the observation arm. 5-year OS with CT improved in 8.6%, which was maintained at 7 years (8.4%).

In 2008, Pignon *et al*. [8] published the LACE study, which included 4584 patients from five studies from the 1990s. Patients were divided into those receiving cisplatin-based CT versus non-CT ones. There was a benefit in OS in favor of CT with a reduction in the risk of death of 11% and an absolute benefit of 3.9% and 5.4% at 3 and 5 years (*P* = 0.005), respectively. The DFS was also in favor of CT with an absolute benefit of 5.8% at 3 and 5 years. In the CT arm, 60% of patients received a dose of 240 mg/m^2^ of cisplatin, and 3–4 cycles were planned. About 64% of the patients in the CT arm and 72% in the control arm received PORT.

In the above-mentioned study [8], the effect of CDDP-VNR was better than when combined with other drugs, without differing significantly. Moreover, there were no important differences regarding the cisplatin dosage, although better results were obtained with doses >300 mg/m^2^. The benefit of CT decreased if the performance status (PS) > 2. The effect of CT was similar in patients with or without PORT. The previously mentioned CT benefit was observed from IB onwards.

A Canadian study [9] focusing on 1032 patients treated with CT documented a median time for adjuvant CT of 8 weeks, and 35% took more than 10 weeks after surgery. No connection between time of CT and OS was found.

Table 1 offers a summary of CT studies pertinent to this review.

**Table 1:** Adjuvant CT in NSCLC

Author/year	Patients n/years	Type CT	Chest RT	Results
International Adjuvant Lung cancer Trial Collaborative Group/2004	1897 (1999–2000)	cisplatin doublets (with vindesine, vinblastine, vinorelbine or etoposide)	30.6% PORT	Absolute OS benefit at 5 years was 4.1%
Winton *et al.*/2005	482 (1994–2001)	CDDP-VNR or observation	No	Better OS in CT arm (94 m vs. 73 m, HR: 0.69, *P=*0.04)
Douillard *et al.*/2006	840 (1994–2000)	CDDP-VNR or observation	28% PORT	Median OS 65.7 months in CT versus 43.7 months in observation
Pignon *et al.*/2008	4584 (from 1995 onwards)	CDDP-VNR	72% PORT	Absolute benefit in CT arm of 3.9% and 5.4% at 3 and 5 years (*P*=0.005)

PORT: Post-operative radiotherapy, OS: Overall survival, CT: Chemotherapy, CDDP-VNR: Cisplatin-vinorelbine

### 1.3. R0

Between 1966 and 1975, Van Houtte [10] screened 175 NSCLC patients with complete resections and no mediastinal involvement, treating them either with PORT or surgery alone, obtaining a lower 5-year OS in the PORT arm (24% vs. 43%). This led to the conclusion that PORT in R0 and pN0 was not advisable. Patients in this study were treated with cobalt.

Between 1989 and 1997, another Italian study [11] also analyzed the use of PORT in complete resections and without lymph node involvement. They assessed a total of 104 patients and found a decrease in local failure after PORT (23% vs. 2.2%) without major toxicity, but without finding statistically significant differences in OS at 5 years’ time.

Patients without mediastinal involvement generally have a lower local recurrence rate, so that the use of PORT is not recommended in these stages if there is no micro or macroscopic involvement.

### 1.4. R1-R2

Between 1 and 17% [12] of lung resections that turn out to be either R1 or R2 incomplete resections, these patients have a higher risk of local recurrence and lower survival rate. PORT is thus recommended to improve local control (LC) and OS in patients where a complete resection is not achieved.

In 2015, Wang *et al*. [12] studied patients with incomplete resections from between 2003 and 2011. These were Stages II–III patients who had received PORT and were listed in the National Cancer Data Base (NCDB). Researchers included patients with doses of 50–74 Gy, so as to leave out palliative patients. In total, they analyzed 3395 patients and divided them between PORT and non-PORT ones. The OS at 5 years was 32.4% versus 23.7% (*P* = 0.001) (HR 0.80, 95%CI, 0.70–0.92) in favor of PORT patients. A dose of 50–70 Gy was associated with a survival benefit, while doses >70Gy displayed similar results to non-PORT patients. They concluded that, in incomplete Stages II–III N0-2 resections, PORT is associated with higher OS. In the R2 cases, radiotherapy (RT) was given as a definitive treatment, not as an adjuvant one.

### 1.5. N0-1

PORT was thought to decrease local failure and increase survival, but the PORT meta-analysis [3] showed that the use of adjuvant radiotherapy decreased OS (from 43% to 30%). This was related to possible pulmonary and cardiac toxicity associated with the techniques used in the 70s. Although toxicities have been considerably reduced with new technological advances, the increase in OS with PORT has not yet been confirmed prospectively.

This meta-analysis was updated by the Cochrane group [13], which screened 2343 patients and found a clear benefit in LC in favor of PORT, with a death increase of 18% in the pN0-1 group. However, this negative effect was not found in pN2.

Higgins *et al*. [14] analyzed 198 pN1 patients between 1995 and 2008, finding out that the risk of local recurrence at 5 years was of 40%, at a distance of 55% with an OS of 33%. Factors associated with local recurrence included invasion of the visceral pleura, number of affected nodes, and videothoracoscopic resection.

1402 pN0-N1 patients resected between 1998 and 2009 were analyzed by López Guerra *et al*. [15] to study locoregional risk factors for recurrence. They concluded that tumor size >2.7 cm, invasion of the visceral pleura, and the extent of surgery were risk factors for local recurrence. Risk of regional recurrence was linked to pN1, invasion of the visceral pleura, and lymphovascular invasion.

In complete resections on pN0-1, the use of PORT is associated to lower survival rates when compared with observation, so it should not be a recommended approach for this subgroup of patients.

### 1.6. N2

Patients with a N2 lymph node station affected after surgery have a higher risk of locoregional failure, something that is correlated with poorer survival.

In 1986, The Lung Cancer Study Group [16] screened 230 patients with NSCLC and mediastinal involvement (II–III), finding out that even if the use of PORT at a dose of 50 Gy decreased the risk of local relapse (1% vs. 41%, *P* = 0.001), it did not have a survival benefit. Such benefit was only found in pN2 patients after performing a subgroup analysis.

Ten years later, the Medical Research Council study [17] was published, assessing the role of PORT on patients operated from pT1-2 pN1-2 with a dose of 40Gy in 15 fractions. No difference in OS was found either, although an improvement in survival was observed in pN2 patients, without being statistically significant.

Similar results were obtained by a Chinese study [18] of 366 pN1-2 patients with complete resections and mediastinal involvement, on whom the use of PORT decreased the risk of local recurrence (12.7% vs. 33.2%, *P* = 0.01), without obtaining a survival benefit.

An analysis of the Surveillance, Epidemiology, and End Results (SEER) was published in 2006 [19], with NSCLC Stages II–III 7465 patients, treated between 1988 and 2002 with either PORT or observation. It demonstrated that the 5-year OS was similar, yet they found a negative effect in patients with pN0-1 was found, as well as a survival benefit in pN2 (5-year OS of 27% vs. 20%, *P* = 0.0036)

The ANITA study [7] focused on the role of adjuvant CT and cisplatin-VNR versus observation in 840 patients with complete resections. Two hundred and thirty-two patients received PORT (45–60 Gy to 2Gy/fr). It was observed that the pN2 who received CT plus PORT had better OS at 5 years than those receiving CT alone (47.4% vs. 34%).

Zou *et al*. [20] analyzed 183 patients from four Chinese centers, comparing PORT plus CT versus CT alone in pN2 patients with complete resections. They obtained higher DFS at 5 years (22.2% vs. 9.3%, *P* = 0.003) and higher OS at 5 years in the combination arm (30.5% vs. 22.2%, *P* = 0.007).

A 2010 meta-analysis [21] demonstrated that adjuvant cisplatin after surgery reduces distant metastases and local failure, observing a benefit in OS at 5 years of 4%, (95% CI 3–6), increasing from 60% to 64%. The comparison of surgery plus RT versus surgery plus RT plus CT also obtained a survival benefit in the CT arm (HR 0·88, 95% CI 0·81–0·97, *P* = 0·009), with an increase in OS at 5 years of 4% (from 29 to 33%).

Corso *et al*. [22] analyzed 30552 NCDB Stage II–IIIA patients from 1998 to 2006. 11.2% of them received PORT with a median dose of 54Gy. In pN0 patients, a negative effect in OS at 5 years was found when compared to surgery alone (37.7% vs. 48%, *P* = 0.001). No differences were found in pN1 patients (34.8 months vs. 39.4 months, *P* = 0.001). A benefit was obtained in terms of survival for pN2 patients (absolute benefit of 6.1% in OS at 5 years) that correlated with the total dose [45–54 Gy yielded better results than doses of 54–60 Gy and better than >60 Gy (41% vs. 32.7% vs. 26.6%)].

A 2015 study [23] analyzed 2115 pN2 patients from the NCDB between 2004 and 2006; all patients in this analysis were treated on linear accelerators in the three dimensional conformal radiation therapy (3DCRT) era. Of those, 43.4% received PORT. They obtained a median overall survival of 42 months in favor of PORT versus 38 months (*P* = 0.048). PORT was associated with longer survival in pN2, even in patients who were receiving CT.

Another study [24] was published in 2015, assessing the impact of PORT with more modern techniques on patients with N2 after surgery and having undergone CT. It included 4483 NCDB patients between 2006 and 2010 and stratified them into PORT (dose> 45 Gy) and non-PORT ones. With a median follow-up of 22 months, it was concluded that the use of PORT versus non-PORT obtained a 3-year OS of 59.3% versus 56.6% and a 5-year OS of 39.3% versus 34.8%, *P* = 0.014. The factors associated with better OS in the multivariate analysis were young age, female sex, urban population, lower Charlson, smaller tumor size, use of CT, at least lobectomy surgery, and use of PORT. In this study, it was concluded that in patients with complete resections and N2 mediastinal involvement, the use of PORT is associated with a benefit in OS when compared to CT alone.

A meta-analysis on PORT [25] divided techniques into modern and old ones, comparing the use of Cobalt versus Linac and concluding that the use of Linac yielded better OS results.

There are other retrospective studies that have shown improved results with new techniques, thanks to smaller volumes and more modern planning techniques, with usual doses of 54 Gy and doses per fraction of 2 Gy.

Francis *et al*. [26] screened the NCDB registry for patients operated with at least one lobectomy and with adjuvant treatments from 2006 to 2012. They divided these into two groups of patients on the basis of anatomic pathology. Group 1 consisted of 747 pT1-3pN2 R0 patients (RT dose: 45–54 Gy) and Group 2 consisted of 277 pT1-3pN0-2 R1-2 patients (45–70 Gy dose). In Group 1, 59% received CT and PORT sequentially, while 41% received concomitant radio-chemotherapy (RTCT). The median OS was in favor of sequential treatment (56.9 months vs. 41.5 months, *P* = 0.019), with a higher risk of death in sequential treatment. In Group 2, 25% of patients underwent sequential treatment and 75% concomitant ones, not finding significant differences between both types of treatment.

In 2016, several papers [27,28] were published questioning the optimal sequence of adjuvant treatments, since they postulated that PORT as the first strategy should be more effective in locoregional control without compromising the OS benefit of CT. Lee *et al*. [29] conducted a retrospective study of 105 pN2 patients who had received PORT as the first adjuvant treatment with or without subsequent CT (4 weeks after completing the RT) due to comorbidities or patient refusal. The 5-year OS was higher in the CT group, 61.3% versus 29.2% (*P* = 0.001). They concluded that PORT as the first treatment does not compromise the results and that the benefit of CT is still noticeable.

Liu *et al*. [30] conducted a systematic review published in 2019 that analyzed the role of PORT in pN2 patients with complete resections. One randomized and 12 retrospective studies were included in the study. Within the PORT group, they observed a benefit in OS [HR = 0.85; 95% confidence interval (CI): 0.79–0.92] and DFS [HR=0.57; 95% CI:0.38–0.85] in patients with multiple N2 or multistation N2 involvement compared with non-PORT ones. No differences were found in OS or DFS between PORT and non-PORT in N2 per single station. No differences were also found regarding age, sex, sizem or histology.

Table 2 offers an overview of PORT in pN2 studies.

**Table 2:** PORT in pN2

Author/year	Patients n/years	Dose RT	Results
Lung Cancer Study Group/1986	230 (1978–1985)	50 Gy	Decreased local relapse (1% vs. 41%, *P* = 0.001). NO survival benefit. After subgroup analysis benefit in pN2 patients.
Stephens *et al.*/1996	308 (1986–1993)	40Gy in 15 fractions	No benefit in OS with PORT
Feng *et al.*/2000	366 (1982–1995)	60 Gy	Decreased local recurrence. No benefit in OS with PORT
Lally *et al.*/2006	7465 (1988–2002)	50 Gy	5-year OS was similar. Negative effect in pN0-1. Survival benefit in pN2 (5-year OS of 27% vs. 20%, *P* = 0.0036)
Corso *et al.*/2015	30552 (1998–2006)	54 Gy	Negative effect in 5 years OS for pN0 (37.7% vs. 48%, *P* < 0.001). No differences in OS in pN1. Benefit in terms of survival for pN2
Mikell *et al.*/2015	2115 (2004–2006)	>35 Gy, <70Gy	Better median OS with PORT (42 months vs. 38 months, *P* = 0.048).
Robinson *et al.*/2015	4483 (2006–2010)	Dose > 45Gy	Better OS with PORT: 3-year OS of 59.3% vs. 55.2% and a 5-year OS of 39.3% vs. 34.8%, *P* = 0.014.
Francis *et al.*/ 2018	1024 (2006–2012)	Group 1: RT dose: 45–54 Gy Group 2: 45–70 Gy dose	The median OS was in favor of sequential treatment (56.9 months vs. 41.5 months, *P* = 0.019)

PORT: Post-operative radiotherapy. OS: Overall survival. RT: Radiotherapy

### 1.7. NSCLC guidelines

The NICE guidelines [31] recommend:

- Considering treatment with adjuvant CT in patients with pT1-4 N1-2M0 NSCLC with good PS.
- Considering adjuvant CT in patients with good PS and pT2b-4NoMo NSCLC with tumors larger than 4 cm.
- The adjuvant CT scheme should be a doublet with cisplatin. Offering adjuvant treatment to patients who benefit from it, considering the benefit-risk.
- The NCCN guidelines [32] recommend:
- Assessing adjuvant CT from a post-operative S.IB (T2aN0) and R0, considering the patient’s risk factors.
- In N2 R0 patients, sequential RT to CT is recommended. The PORT dose should be 50–54 Gy to 2 Gy/fr and an increase to 60–66 Gy could be performed in the event of a positive margin or extracapsular involvement.
- In R1–R2 patients, adjuvant CT and RT are recommended concomitantly if the patient’s characteristics allow it.

### 1.8. Definition of volumes

The definition of volumes for adjuvant treatment is based on a study [33], in which 17 radiation oncologists contoured their clinical target volumes (CTVs) in two patient models with the same program. The CTV of the operated tumor should include the affected lymph node regions, the bronchial stump, and the ipsilateral hilum. The ganglion areas must also include the superior and inferior regions, due to their frequent involvement. Table 3 describes the limits according to the affected lymph node region [34]. The CTV should be given a margin of 0.5 cm in the anteroposterior and lateral regions and of 1 cm in the cranio-caudal one.

**Table 3:** Radiation limits regarding involved node station

Involved lymph node station	Lymph node levels included in CTV	Upper limit	Lower limit
1–2	1–2, 4, 10 ipsilateral and 7	1 cm over the sternal notch	4 cm below the carina
3–4	3, 4, 10 ipsilateral and 7.	Sternal notch	
4	2, 4, 10 ipsilateral and 7	Sternal notch	
5	2 years 4 L, 5, 6, and 7	Upper aortic arch	
6		Sternal notch	
7	4R years 7 if right tumor 4L, 5, 6 years 7 if left tumor	Upper aortic arch	5 cm below the carina
8	4R, 7 yard 8 if right tumor 4 L, 5, 6, 7 y 8 if left tumor.		Gastroesophageal union

In 2013 Olszyna *et al*. [35] retrospectively analyzed 80 patients with incomplete resections between 2002 and 2011, divided according to the presence or absence of extracapsular extension (ECE). They performed elective nodal irradiation (ENI), including the bronchial stump, affected lymph node station, and lymph node stations with higher probability of microinvasion in the CTV (e.g., the ipsilateral hilum, paratracheal, subcarin and prevascular ones, and, on the left side, region 5).The CTV was given a one-centimeter margin. The dose was 54–56 Gy, increasing up to 60 Gy in the case of N2 with ECE. In cases of incomplete resection of the thoracic wall, this was the only irradiated area, up to 60 Gy. Median OS without ECE was 31 months versus 24 months with ECE (*P* = 0.43).

### 1.9. Awaiting publication

- Lung ART trial [36]: A Phase-III study from the Goustave-Roussy Institute, comparing treatment with modern-technique PORT versus observation in patients with NSCLC resections. The primary goal is DFS. As secondary ones, toxicity, SG and local control. At ESMO 2020 Cecile Le Pechoux presented the preliminary data, including 252 patients in the PORT arm versus 249 in the control arm. No improvement at 3-years DFS was found (43.8% vs. 47.1%, HR: 0.85, *P* = 0.16), nor OS (66.5% in PORT arm vs. 68.5% in non-PORT arm). A benefit was found in local relapse (46.1% vs. 25%), but associated to an increase in patient death (5.3% vs. 14.6%) in the RT arm, as a result of cardio-pulmonary toxicity (16.2%).
- A Chinese study called NCT 00880971 [37]: A Chinese Phase III study comparing the role of PORT after CT versus only CT in pN2 patients with complete resections. This study was designed to assess the role of PORT in survival and local relapse terms. 364 patients from between 2009 and 2017 were stratified into those receiving PORT (50 Gy/25 fractions, with 3D/IMRT) versus observation. The end point was DFS and the secondary ones, OS, DF, locoregional recurrence, DF metastasis, and toxicity. The results presented at the 2019 World Conference on Lung Cancer demonstrate a 3-year DFS of 42.7% in the PORT arm versus 34.5% in observation, median DFS of 26.5 versus 22.7 months (HR:0.85, *P* = 0.10).

## 2. Adjuvancy in SCLC

### 2.1. Introduction

SCLC (small-cell lung cancer) represents 15% of all lung tumors. About 30% are diagnosed as limited disease (LD), while 70% are already extensive disease (ED). Only 5% of patients are in Stage I at diagnosis. The prognosis is related to the stage in which the disease is found. There are authors who say that it should always be treated as a systemic disease, even when it seems to be localized [38,39].

The fundamental treatment in any of the stages is CT based on platinum-VP16, in LD in combination with concomitant RT, and initiation as soon as possible.

This type of tumor is characterized by a rapid doubling time, with early dissemination, high sensitivity to both the CT and RT treatments, together with subsequent resistance to the abovementioned treatments [40].

There are no randomized studies comparing surgery alone versus surgery plus CT versus RTCT.

### 2.2. The role of surgery

Fox *et al*. [41] carried out a prospective study that included 144 patients, grouping them into surgery or RT. At 10 years, no survivors were found in the surgery arm, while there did exist some in the RT arm. Median survival for the surgery arm was 199 days, while for the RT arm it was 300 days.

Schreider *et al*. [42] screened 14,179 patients from the SEER database between 1988 and 2002 with a diagnosis of SCLC, of whom 863 were cT1-2 who had undergone surgery. Surgical patients had better survival rates than those who had not underwent surgery, with a median survival of 42 months versus 15 months. The 5-year OS of surgical patients was 45% in localized disease patients and 26% in patients with regional disease. This study presents a number of limitations given the fact that it is a retrospective study. Among them, we could mention that it is impossible to know which patients received PORT, the patients’ PS or the state of the margins so that the results obtained should be interpreted with caution.

Another SEER study [43] compared SCLC patients between 1988 and 2004 who had undergone surgery plus PORT versus surgery alone. Adjuvant CT was chosen for all patients. OS at 5 years for surgery plus PORT patients was 57% versus surgery alone ones, which was of 50% (*P* = 0.09) In 2017, a study [44] analyzing 29,994 patients with SCLC from the NCDB was published. Out of these, 2089 underwent surgery plus CT plus RT providing that they were pN+ and were matched with non-surgical patients (CT ± RT). In patients with limited cN0 disease, OS was higher in surgical patients (38 months vs. 22 months). This benefit was only found in R0 patients with lobectomies and adjuvant CT.

In recent decades there have been several studies on surgery in limited stages, obtaining a 5-year survival for Stage I after surgery of 40–60% [45-49].

### 2.3. CT

In the 1990s, several studies suggested the benefit of CT after surgery [50-53]. 4 prospective Phase II studies exist, assessing the effects of surgery plus CT on LD with different CT regimens, with or without PORT.

An Italian Phase II study [54] assessed 42 cT1-3N0 SCLC patients between 1980 and 1986 who had received surgery plus CT ×6 (cyclophosphamide, epirubicin, VP16) without PCI (prophylactic cranial irradiation) or chest RT. They obtained a 5-year OS of 36% and a median survival of 32.7 months. Tumor size was the only factor found to influence survival. Treatment was well tolerated, with acceptable toxicity.

Another Phase II study [55] from The Lung Cancer Study Group of the International Society of Chemotherapy (ISC-LCSG) assessed the role of surgery plus CT ×6 (cyclophosphamide, doxorubicin, vincristine) + PCI in 183 T1-2N0 patients. A 30-month survival of 63% for T1 and 37% for T2 was achieved.

Rea *et al*. [56] analyzed the long-term results of 104 patients from between 1981 and 1995 who had undergone surgery plus CT ×6 plus RT. Fifty-one patients underwent surgery plus CT plus chest RT and 53 underwent CT plus surgery plus chest RT. The 5-year OS was 32%. As the stage increased, the OS decreased.

A Japanese Phase II study [57] evaluated 62 patients between 1991 and 1996 with SCLC, complete resections and four cycles of adjuvant platinum-VP16-based CT. The 3-year OS was 61%. Local recurrence was 10% and distance recurrence was 34%.

In April 2016, Yang *et al*. [58] published an analysis of NCDB patients with SCLC cT1-2N0, who had been operated with adjuvant CT between 2003 and 2011, with or without PCI. Out of these, 954 were patients with complete surgery and 566 were patients with adjuvant, CT, RTCT, and RT alone. Compared to surgery alone, adjuvant CT with or without RT was associated with an improvement in OS. Advanced age or larger tumor size were factors linked to worse survival. This study does neither describe the type of CT (doublet with platinum) nor the volume of planning for RT. It does not specify as well which patients received chest RT or PCI.

The same research group from the previous study analyzed 4729 NCDB patients [59] from between 2003 and 2011 with cT1-2N0 SCLC. Out of these, 681 patients underwent surgery plus CT plus RT and 1620 underwent concomitant RTCT. After performing the propensity score-matched for the analysis, they concluded that surgery was associated with a longer survival than RTCT, a 5-year OS of 48% and 30%, respectively. In the absence of prospective studies, this review recommends multimodal treatment with surgery plus CT, stating that RT should be taken into consideration.

There is an on-going Japanese Phase III JCOG1205/1206 study comparing patients with resected high-grade neuroendocrine tumors (including SCLC) adjuvant with platinum-VP16 versus irinotecan-platinum, where the main objective is OS.

Table 4 offers an overview of CT studies.

**Table 4:** Overview of CT in SCLC

Author/year	Patients n/years	Stage	Type CT	PCI/Chest RT	Results
Macchiarini *et al.*/1991	42 (1980–1986)	cT1-3N0	Cyclophosphamide, epirubicin, VP16 x6	No	5-year OS 36% Median survival: 32.7 months
Karrer *et al.*/1995	183	cT1-2N0	Cyclophosphamide, doxorubicin, vincristine x6	PCI	30-month survival of 63% for T1 and 37% for T2
Rea *et al.*/1998	104 (1981–1995)	I-II-III	Cyclophosphamide, doxorubicin, vincristine x4-6 (1981–1988) Platinum-VP16-epirubicin (1988–1995)	PCI (17 Gy) RT chest (40 Gy/20 fractions)	5-year OS was 32%. Survival data were 52.2%, 30% and 15.3% for Stage I, II, and III, respectively (*P*<0.001).
Tsuchiya *et al.*/2005	62 (1991–1996)	I-II-IIIA	Platinum-VP16 x4	No	3-year OS 61% Local failure was 10%. Locoregional recurrence more frequently in Stage IIIA disease. Brain metastasis was found in 15% of the patients.
Yang *et al.*/2016	954 (2003–2011)	cT1-2N0	No specific adjuvant chemotherapy regimens	PCI RT chest	CT associated improved OS. 5-year OS (52.7% vs. 40.4%, *P=*0.01.

RT: Radiotherapy, OS: Overall survival, CT: Chemotherapy, PCI: Profilactic cranial irradiation

### 2.4. PORT

Wong *et al*. [60] analyzed 3017 SCLC patients from the NCBD from between 1998 and 2011 with complete resections and adjuvant (or not) PORT (minimum dose 45 Gy). The 5-year OS was lower in the RT group (33.9% vs. 40.6%, *P* = 0.005). In the analysis by subgroups, pN2 patients did benefit from PORT (OS 29% vs. 18.6%, *P* = 0.001).

Another study [61] examines the role of PORT in 3101 NCBD SCLC patients operated between 2004 and 2013 who had undergone lobectomy, pneumonectomy, or sublobar resections. The patients who received PORT were the youngest ones, with advanced T tumors and lymph node involvement, having undergone sublobar resection or with positive margins. Adjuvant mediastinal RT was associated with a lower risk of death in pN1 (HR 0.79; 95% CI: 0.63–1.00, *P* = 0.05) and in pN2 (HR0.60; 95% CI: 0.48–0.75, *P* = 0.0001). However, it was not associated with better survival in patients without lymph node involvement. Nevertheless, it improved survival in patients treated with sublobar resection (HR 0.72, 95% CI: 0.57–0.90, *P* = 0.004).

In 2019 Engelhardt *et al*. [62] retrospectively analyzed patients with SCLC with complete pT1-2N0 resections from the NCDB database and stratified them into those who had received PORT and those who had not. No significant differences were found in performing adjuvant PORT.

The opinion of 13 European experts [63] regarding whether to perform PORT or PCI in SCLC resections has been recently published. It concludes that PORT and PCI should be performed for resected patients with lymph node involvement. For patients of advanced age or without lymph node involvement, PORT or PCI are not recommended.

Table 5 offers a summary of PORT studies in SCLC.

**Table 5:** PORT in SCLC

Author/year	Patients n/years		Dose PORT	Results
Wong *et al.*/2016	3017 (1998–2011) NCDB	SCLC R0: PORT or not	45 Gy	The 5-year OS worse in RT (33.9% vs. 40.6%, *P* = 0.005). Analysis by subgroups: benefit in pN2 with PORT (OS 29% vs. 18.6%, *P* = 0.001).
Wakeam *et al.*/2017	3101 NCDB (2004–2013)	Role of PORT in lobectomy, pneumonectomy or sublobar resections.	>40 Gy	RT: lower risk of death in pN1 (*P* = 0.05) and pN2 (*P* = 0.0001). Better survival in patients with sublobar resection (*P* = 0.004).
Engelhardt *et al.*/2019	1617 NCDB (2004–2014)	PORT or not	No specific dose RT	No differences with PORT. Median OS surgery alone 62.2 months versus surgery + RT 43.8 months; *P* = 0.14.

PORT: Post-operative radiotherapy, OS: Overall survival, RT: Radiotherapy, SCLC: Small-cell lung cancer, NCDB: National cancer database

### 2.5. SCLC guidelines

The NICE guidelines [31] consider surgery if SCLC cT1-2aN0M0.

The NCCN guidelines [64] offer similar recommendations:

- Considering surgery in T1-2N0 SCLC with correct mediastinal study. As adjuvant treatment in both N0-N+, adjuvant CT should be considered, preferably cisplatin-etoposide, or carboplatin-etoposide.
- Post-operative RT is recommended for pN2 and should be taken into consideration in pN1, concomitant, or sequential to CT.

### 2.6. Definition of volumes

When PORT is administered, the CTV should include the affected mediastinal region, the ipsilateral hilar region, the bronchial stump, and the subcarinal station [65].

If neoadjuvant CT is to be performed, previously affected lymph node stations should be included, even if there is down staging.

If an adequate lymphadenectomy is not obtained in the surgery and there is no mediastinal involvement, each patient could be assessed individually, choosing one of the following options: Either close monitoring with CT every 3 months (preferably), or PORT to all lymph node stations at risk depending of the location of the primary tumor.

## 3. Discussion

The role of PORT in NSCLC has always been a controversial issue due to the negative studies published to date. It was thought that the excess toxicity observed could be explained by the old irradiation techniques, the large volumes of treatment and the high doses and fractionations used.

PORT should not be used in complete pN0-1 resections due to published studies recording deleterious effects.

It was expected that the role of PORT in pN2 would be solidly ascertained, but after the preliminary results of the LUNGART presented at ESMO 2020, this remains in doubt. No benefit in DFS was obtained in patients with complete pN2 resections treated with PORT versus observation alone, demonstrating instead greater cardiopulmonary toxicity.

The role of PORT should be performed individually and should not be considered a standard treatment in pN2 NSCLC.

The role of PORT in SCLC is less frequent due to the low frequency of potentially surgical early-stage tumors. PORT and PCI should be considered in pN1-2 after systemic treatment based on platinum-etoposide.

### Conflict of Interest

The authors declare no conflict of interest related to the submitted work in accordance with the ICMJE guidelines.

## References

[1] Péchoux CL, Mercier O, Belemsagha D, Bouaita R, Besse B, Fadel E (2013). Role of Adjuvant Radiotherapy in Completely Resected Non-Small-Cell Lung Cancer. EJC Suppl.

[2] Chemotherapy in Non-Small Cell Lung Cancer:A Meta-Analysis Using Updated Data on Individual Patients from 52 Randomised Clinical Trials (1995). Non-Small Cell Lung Cancer Collaborative Group. BMJ.

[3] Postoperative Radiotherapy in Non-Small-Cell Lung Cancer:Systematic Review and Meta-Analysis of Individual Patient Data from Nine Randomised Controlled Trials (1998). PORT Meta-Analysis Trialists Group. Lancet.

[4] Rami-Porta R, Wittekind C, Goldstraw P, International Association for the Study of Lung Cancer (IASLC) Staging Committee (2005). Complete Resection in Lung Cancer Surgery:Proposed Definition. Lung Cancer.

[5] Arriagada R, Bergman B, Dunant A, Le Chevalier T, Pignon JP, Vansteenkiste J (2004). Cisplatin-Based Adjuvant Chemotherapy in Patients with Completely Resected Non-Small-Cell Lung Cancer. N Engl J Med.

[6] Butts CA, Ding K, Seymour L, Twumasi-Ankrah P, Graham B, Gandara D (2010). Randomized Phase III Trial of Vinorelbine Plus Cisplatin Compared with Observation in Completely Resected Stage IB and II Non-Small-Cell Lung Cancer: Updated Survival Analysis of JBR-10. J Clin Oncol.

[7] Douillard JY, Rosell R, de Lena M, Carpagnano F, Ramlau R, Gonzáles-Larriba JL (2006). Adjuvant Vinorelbine Plus Cisplatin Versus Observation in Patients with Completely Resected Stage IB-IIIA Non-Small-Cell Lung Cancer (Adjuvant Navelbine International Trialist Association [ANITA]):A Randomised Controlled Trial. Lancet Oncol.

[8] Pignon JP, Tribodet H, Scagliotti GV, Douillard JY, Shepherd FA, Stephens RJ (2008). Lung Adjuvant Cisplatin Evaluation:A Pooled Analysis by the LACE Collaborative Group. J Clin Oncol.

[9] Booth CM, Shepherd FA, Peng Y, Darling G, Li G, Kong W (2013). Time to Adjuvant Chemotherapy and Survival in Non-Small Cell Lung Cancer:A Population-Based Study. Cancer.

[10] van Houtte P, Rocmans P, Smets P, Goffin JC, Lustman-Maréchal J, Vanderhoeft P (1980). Postoperative Radiation Therapy in Lung Caner:A Controlled Trial after Resection of Curative Design. Int J Radiat Oncol Biol Phys.

[11] Trodella L, Granone P, Valente S, Valentini V, Balducci M, Mantini G (2002). Adjuvant Radiotherapy in Non-Small Cell Lung Cancer with Pathological Stage I:Definitive Results of a Phase III Randomized Trial. Radiother Oncol.

[12] Wang EH, Corso CD, Rutter CE, Park HS, Chen AB, Kim AW (2015). Postoperative Radiation Therapy is Associated with Improved Overall Survival in Incompletely Resected Stage II and III Non-Small-Cell Lung Cancer. J Clin Oncol.

[13] Burdett S, Rydzewska L, Tierney J, Fisher D, Parmar MK, Arriagada R (2003). Postoperative Radiotherapy for Non-Small Cell Lung Cancer. Cochrane Database Syst Rev.

[14] Higgins KA, Chino JP, Berry M, Ready N, Boyd J, Yoo DS (2012). Local Failure in Resected N1 Lung Cancer:Implications for Adjuvant Therapy. Int J Radiat Oncol Biol Phys.

[15] Guerra JL, Gomez DR, Lin SH, Levy LB, Zhuang Y, Komaki R (2013). Risk Factors for Local and Regional Recurrence in Patients with Resected N0-N1 Non-Small-Cell Lung Cancer, with Implications for Patient Selection for Adjuvant Radiation Therapy. Ann Oncol.

[16] Lung Cancer Study Group (1986). Effects of Postoperative Mediastinal Radiation on Completely Resected Stage II and Stage III Epidermoid Cancer of the Lung. N Engl J Med.

[17] Stephens RJ, Girling DJ, Bleehen NM, Moghissi K, Yosef HM, Machin D (1996). The Role of Post-Operative Radiotherapy in Non-Small-Cell Lung Cancer:A Multicentre Randomised Trial in Patients with Pathologically Staged T1-2, N1-2, M0 Disease. Medical Research Council Lung Cancer Working Party. Br J Cancer.

[18] Feng QF, Wang M, Wang LJ, Yang ZY, Zhang YG, Zhang DW (2000). A Study of Postoperative Radiotherapy in Patients with Non-Small-Cell Lung Cancer:A Randomized Trial. Int J Radiat Oncol Biol Phys.

[19] Lally BE, Zelterman D, Colasanto JM, Haffty BG, Detterbeck FC, Wilson LD (2006). Postoperative Radiotherapy for Stage II or III Non-Small-Cell Lung Cancer Using the Surveillance, Epidemiology, and End Results Database. J Clin Oncol.

[20] Zou B, Xu Y, Li T, Li W, Tang B, Zhou L (2010). A Multicenter Retrospective Analysis of Survival Outcome Following Postoperative Chemoradiotherapy in Non-Small-Cell Lung Cancer Patients with N2 Nodal Disease. Int J Radiat Oncol Biol Phys.

[21] Arriagada R, Auperin A, Burdett S, Higgins JP, Johnson DH, NSCLC Meta-Analyses Collaborative Group (2010). Adjuvant Chemotherapy, With or Without Postoperative Radiotherapy, in Operable Non-Small-Cell Lung Cancer:Two Meta-Analyses of Individual Patient Data. Lancet.

[22] Corso CD, Rutter CE, Wilson LD, Kim AW, Decker RH, Husain ZA (2015). Re-Evaluation of the Role of Postoperative Radiotherapy and the Impact of Radiation Dose for Non-Small-Cell Lung Cancer Using the National Cancer Database. J Thorac Oncol.

[23] Mikell JL, Gillespie TW, Hall WA, Nickleach DC, Liu Y, Lipscomb J (2015). Postoperative Radiotherapy is Associated with Better Survival in Non-Small Cell Lung Cancer with Involved N2 Lymph Nodes:Results of an Analysis of the National Cancer Data Base. J Thorac Oncol.

[24] Robinson CG, Patel AP, Bradley JD, DeWees T, Waqar SN, Morgensztern D (2015). Postoperative Radiotherapy for Pathologic N2 Non-Small-Cell Lung Cancer Treated with Adjuvant Chemotherapy:A Review of the National Cancer Data Base. J Clin Oncol.

[25] Billiet C, Decaluwé H, Peeters S, Vansteenkiste J, Dooms C, Haustermans K (2014). Modern Post-Operative Radiotherapy for Stage III Non-Small Cell Lung Cancer May Improve Local Control and Survival:A Meta-Analysis. Radiother Oncol.

[26] Francis S, Orton A, Stoddard G, Tao R, Hitchcock YJ, Akerley W (2018). Sequencing of Postoperative Radiotherapy and Chemotherapy for Locally Advanced or Incompletely Resected Non-Small-Cell Lung Cancer. J Clin Oncol.

[27] Selek U, Chang JY (2016). Optimal Sequencing of Postoperative Radiotherapy and Chemotherapy in IIIA-N2 Non-Small Cell Lung Cancer. J Thorac Dis.

[28] Kepka L, Socha J, Rucinska M, Wasilewska-Tesluk E, Komosinska K (2016). Sequencing Postoperative Radiotherapy and Adjuvant Chemotherapy in Non-Small Cell Lung Cancer:Unanswered Questions on the Not Evidence-Based Approach. J Thorac Dis.

[29] Lee HW, Noh OK, Oh YT, Choi JH, Chun M, Kim HI (2016). Radiation Therapy-First Strategy After Surgery With or Without Adjuvant Chemotherapy in Stage IIIA-N2 Non-Small Cell Lung Cancer. Int J Radiat Oncol Biol Phys.

[30] Liu T, Mu Y, Dang J, Li G (2019). The Role of Postoperative Radiotherapy for Completely Resected pIIIA-N2 Non-Small Cell Lung Cancer Patients with Different Clinicopathological Features:A Systemic Review and Meta-Analysis. J Cancer.

[31] NICE Guidelines (2019). https://www.nice.org.uk/guidance/ng122.

[32] NCCN Guidelines, v8-2020 https://www.nccn.org/professionals/physician_gls/pdf/nscl.pdf.

[33] Spoelstra FO, Senan S, Le Péchoux C, Ishikura S, Casas F, Ball D (2010). Variations in Target Volume Definition for Postoperative Radiotherapy in Stage III Non-Small-Cell Lung Cancer:Analysis of an International Contouring Study. Int J Radiat Oncol Biol Phys.

[34] Gómez A, González JA, Couñago F, Vallejo C, Casas F, de Dios NR (2016). Evidence-Based Recommendations of Postoperative Radiotherapy in Lung Cancer from Oncologic Group for the Study of Lung Cancer (Spanish Radiation Oncology Society). Clin Transl Oncol.

[35] Olszyna-Serementa M, Socha J, Wierzchowski M, Kępka L (2013). Patterns of Failure after Postoperative Radiotherapy for Incompletely Resected (R1) Non-Small Cell Lung Cancer:Implications for Radiation Target Volume Design. Lung Cancer.

[36] Le Pechoux C, Pourel N, Barlesi F, Faivre-Finn C, Lerouge D, Zalcman G (2020). Phase III Study Comparing Post-Operative Conformal Radiotherapy to No Post-Operative Radiotherapy in Patients With Completely Resected Non-Small Cell Lung Cancer and Mediastinal N2 Involvement [Lung ART] ESMO.

[37] Hui Z, Men Y, Hu C (2019). Phase III Comparison of Postoperative 3D Conform Radiotherapy after Chemotherapy and Chemotherapy Alone in Patients with Completely Resected Stage IIIA(N2) Non-Small Cell Lung Cancer. J Thorac Oncol.

[38] Rossi A (2016). Adjuvant Therapy in Early-Stage Small-Cell Lung Cancer:What's New?. Transl Cancer Res.

[39] Valliers E (2020). Role of Surgery in Multimodality Therapy for Small Cell Lung Cancer. https://www.uptodate.com.

[40] Jackman DM, Johnson BE (2005). Small-Cell Lung Cancer. Lancet.

[41] Fox W, Scadding JG (1973). Medical Research Council Comparative Trial of Surgery and Radiotherapy for Primary Treatment of Small-Celled or Oat-Celled Carcinoma of Bronchus. Ten-Year Follow-Up. Lancet.

[42] Schreiber D, Rineer J, Weedon J, Vongtama D, Wortham A, Kim A (2010). Survival Outcomes with the Use of Surgery in Limited-Stage Small Cell Lung Cancer:Should Its Role be Re-Evaluated?. Cancer.

[43] Yu JB, Decker RH, Detterbeck FC, Wilson LD (2010). Surveillance Epidemiology and End Results Evaluation of the Role of Surgery for Stage I Small Cell Lung Cancer. J Thorac Oncol.

[44] Wakeam E, Acuna SA, Leighl NB, Giuliani ME, Finlayson SR, Varghese TK (2017). Surgery Versus Chemotherapy and Radiotherapy For Early and Locally Advanced Small Cell Lung Cancer:A Propensity-Matched Analysis of Survival. Lung Cancer.

[45] Inoue M, Miyoshi S, Yasumitsu T, Mori T, Iuchi K, Maeda H (2000). Surgical Results for Small Cell Lung Cancer Based on the New TNM Staging System. Thoracic Surgery Study Group of Osaka University, Osaka, Japan. Ann Thorac Surg.

[46] Rostad H, Naalsund A, Jacobsen R, Strand TE, Scott H, Strøm EH (2004). Small Cell Lung Cancer in Norway. Should More Patients Have Been Offered Surgical Therapy?. Eur J Cardiothorac Surg.

[47] Brock MV, Hooker CM, Syphard JE, Westra W, Xu L, Alberg AJ (2005). Surgical Resection of Limited Disease Small Cell Lung Cancer in the New Era of Platinum Chemotherapy:Its Time has Come. J Thorac Cardiovasc Surg.

[48] Lim E, Belcher E, Yap YK, Nicholson AG, Goldstraw P (2008). The Role of Surgery in the Treatment of Limited Disease Small Cell Lung Cancer:Time to Reevaluate. J Thorac Oncol.

[49] Schneider BJ, Saxena A, Downey RJ (2011). Surgery for Early-Stage Small Cell Lung Cancer. J Natl Compr Canc Netw.

[50] Karrer K, Shields TW, Denck H, Hrabar B, Vogt-Moykopf I, Salzer GM (1989). The Importance of Surgical and Multimodality Treatment for Small Cell Bronchial Carcinoma. J Thorac Cardiovasc Surg.

[51] Merkle NM, Mickisch GH, Kayser K, Drings P, Vogt-Moykopf I (1986). Surgical Resection and Adjuvant Chemotherapy for Small Cell Carcinoma. Thorac Cardiovasc Surg.

[52] Osterlind K, Hansen M, Hansen HH, Dombernowsky P (1986). Influence of Surgical Resection Prior to Chemotherapy on the Long-Term Results in Small Cell Lung Cancer.
A Study of 150 Operable Patients. Eur J Cancer Clin Oncol.

[53] Shepherd FA, Ginsberg RJ, Feld R, Evans WK, Johansen E (1991). Surgical Treatment for Limited Small-Cell Lung Cancer. The University of Toronto Lung Oncology Group Experience. J Thorac Cardiovasc Surg.

[54] Macchiarini P, Hardin M, Basolo F, Bruno J, Chella A, Angeletti CA (1991). Surgery Plus Adjuvant Chemotherapy for T1-3N0M0 Small-Cell Lung Cancer. Rationale for Current Approach. Am J Clin Oncol.

[55] Karrer K, Ulsperger E (1995). Surgery for Cure Followed by Chemotherapy in Small Cell Carcinoma of the Lung. For the ISC-Lung Cancer Study Group. Acta Oncol.

[56] Rea F, Callegaro D, Favaretto A, Loy M, Paccagnella A, Fantoni U (1998). Long Term Results of Surgery and Chemotherapy in Small Cell Lung Cancer. Eur J Cardiothorac Surg.

[57] Tsuchiya R, Suzuki K, Ichinose Y, Watanabe Y, Yasumitsu T, Ishizuka N (2005). Phase II Trial of Postoperative Adjuvant Cisplatin and Etoposide in Patients with Completely Resected Stage I-IIIa Small Cell Lung Cancer:The Japan Clinical Oncology Lung Cancer Study Group Trial (JCOG9101). J Thorac Cardiovasc Surg.

[58] Yang CF, Chan DY, Speicher PJ, Gulack BC, Wang X, Hartwig MG (2016). Role of Adjuvant Therapy in a Population-Based Cohort of Patients with Early-Stage Small-Cell Lung Cancer. J Clin Oncol.

[59] Yang CF, Chan DY, Yerokun B, Wang X, Tong BC, Dámico T (2016). Surgery Versus Optimal Medical Management of Early-Stage Small Cell Lung Cancer. J Clin Oncol.

[60] Wong AT, Rineer J, Schwartz D, Schreiber D (2016). Assessing the Impact of Postoperative Radiation Therapy for Completely Resected Limited-Stage Small Cell Lung Cancer Using the National Cancer Database. J Thorac Oncol.

[61] Wakeam E, Giuliani M, Leighl NB, Finlayson SR, Varghese TK, Darling GE (2017). Indications for Adjuvant Mediastinal Radiotherapy in Surgically Resected Small Cell Lung Cancer. Ann Thorac Surg.

[62] Engelhardt KE, Coughlin JM, DeCamp MM, Denlinger CE, Meyerson SL, Bharat A (2019). Survival after Adjuvant Radiation Therapy in Localized Small Cell Lung Cancer Treated with Complete Resection. J Thorac Cardiovasc Surg.

[63] Putora PM, de Ruysscher D, Glatzer M, Widder J, van Houtte P, Troost EG (2020). The role of postoperative thoracic radiotherapy and prophylactic cranial irradiation in early stage small cell lung cancer:Patient selection among ESTRO experts. Radiother Oncol.

[64] NCCN Guidelines, v1 (2021). https://www.nccn.org/professionals/physician_gls/pdf/sclc.pdf?.

[65] Le Pechoux C, Faivre-Finn C, Ramella S, McDonald F, Manapov F, Putora PM (2020). ESTRO ACROP Guidelines for Target Volume Definition in the Thoracic Radiation Treatment of Small Cell Lung Cancer. Radiother Oncol.

